# Nutrition Education by a Registered Dietitian Improves Dietary Intake and Nutrition Knowledge of a NCAA Female Volleyball Team

**DOI:** 10.3390/nu4060506

**Published:** 2012-06-08

**Authors:** Melinda W. Valliant, Heather Pittman Emplaincourt, Rachel Kieckhaefer Wenzel, Bethany Hilson Garner

**Affiliations:** Nutrition and Hospitality Management, University of Mississippi, 222 Lenoir Hall, University, MS 38677, USA; Email: hfpittman@gmail.com (H.P.E.); rachelewenzel@gmail.com (R.K.W.); bhilson@gmail.com (B.H.G.)

**Keywords:** female, athletes, energy, carbohydrate, nutrition, knowledge

## Abstract

Eleven female participants from a NCAA Division I volleyball team were evaluated for adequate energy and macronutrient intake during two off-seasons. Total energy and macronutrient intake were assessed by food records and results were compared against estimated needs using the Nelson equation. Dietary intervention was employed regarding the individual dietary needs of each athlete as well as a pre- and post-sports nutrition knowledge survey. Post dietary intervention, total energy, and macronutrient intake improved, as well as a significant improvement in sports nutrition knowledge (*p* < 0.001). Nutrition education is useful in improving dietary intake and nutrition knowledge of female athletes.

## 1. Introduction

Female participation in sports dramatically increased following the passage of the Education Amendment of 1972, also known as “Title IX,” and its reinforcement in the 1988 Civil Rights Restoration Act [1]. Since Title IX, the number of female athletes has risen from nearly 32,000 in 1971 to more than 150,000 in 2000 [2]. This rise in female athletes has contributed to an extreme amount of support for volleyball, and it is evidenced by the 603 NCAA Division I, II, and III women’s volleyball teams in 1981 that nearly doubled to 1015 teams in 2008 [1]. As a result of this increase in support, a considerable amount of research has been attributed to understanding the female physiology and major nutritional concerns of female athletes. 

Currently, a vast amount of research is available on nutrient intake among female athletes [3,4,5,6,7,8,9]. However, there is little research addressing sport-specific nutritional needs, especially those of volleyball players. Volleyball is a high intensity game requiring speed and large muscle groups for actions such as jumping, spiking, blocking, and retrieving the ball. Moreover, if a player were to spend an equal amount of time between the front and back courts, these high intensity actions can occur in shorts bouts every 30 s of play over a total period of 30 min to 180 min [10]. Consequently, during the off season female volleyball players have been reported to have total energy expenditures as high as 2815 ± 306 [11]. It is essential for an athlete expending energy at these high levels to consume adequate calories, yet a number of studies indicate that female athletes fail to meet these energy demands while training and competing [5,7,8,11,12,13]. According to the American College of Sports Medicine, adequate calorie intake is needed to maintain lean tissue mass, immune and reproductive function, and optimal athletic performance [3]. When calorie intake is inadequate, the body will use fat and lean tissue mass as fuel, and as a result, muscle mass will be lost and strength and endurance will be compromised [3]. 

Achieving energy balance is a critical component to meeting adequate energy requirements. Energy balance occurs when energy intake equals energy expended. Energy expenditure is influenced by a number of factors including the type of exercise, duration and intensity of exercise, age, gender, body size, fat-free mass (FFM), and nutritional status prior to exercise [14]. The Recommended Dietary Allowance (RDA) for normally active people states that individuals should consume energy at a rate of 37 to 41 kcal/kg of body weight [3]. For female athletes who engage primarily in resistance training, the energy requirements may be as high as 39 to 44 kcal/kg of body weight to support high levels of fat free mass and thus to maintain body weight [15]. 

In addition to meeting energy requirements, macronutrients must be consumed in adequate amounts to sufficiently replenish glycogen stores. An important factor impacting muscle glycogen storage is carbohydrate consumption [16]. Many female athletes restrict their carbohydrate intake and, as a result, do not meet the RDA to maintain muscle glycogen stores [6,17]. Athletes are generally recommended to consume 60% of energy from carbohydrates assuming that energy intake is adequate to meet needs. Moreover, a range of 6 to 10 g of carbohydrates per kilogram of body weight per day is recommended to meet optimal muscle glycogen storage [14].

All of the foregoing research explains why adequate energy intake is necessary to achieve optimal performance. However, despite this readily available research, it is a well known fact that low energy availability is a prevailing issue among female athletes across many sports. In addition, female athletes have demonstrated a limited working knowledge of sports nutrition [18,19]. Many researchers have suggested that athletes as well as coaches will benefit from nutrition education and counseling regarding performance and prevention of health concerns [4,6,8,18,19,20]. Therefore, to gain a better understanding of the foregoing issues, a preliminary investigation of a 2009 NCAA Division I women’s volleyball team was conducted. The study was performed during the 2009 off-season, and throughout the study, no nutritional intervention was employed. This investigation revealed participants’ diets were inadequate to meet their daily caloric needs [21]. Thus, the purpose of this paper is to conduct an evaluation of dietary intake, nutrition knowledge, and whether education improves dietary intake of collegiate female volleyball players. 

## 2. Experimental Section

### 2.1. Experimental Approach to the Problem

The present study was designed to conduct an evaluation of dietary intake, nutrition knowledge, and whether education improves dietary intake of collegiate female volleyball players over the course of two off-seasons. The first off season (non-intervention), baseline measures of dietary intake was assessed using 3-day food records that were obtained at the beginning and end of the off-season, with participants receiving no education regarding their diet. During the second off season (intervention), 3-day food records were obtained once a month for a total of four months. Participants received individualized dietary education with the intent to increase each student-athlete’s individual knowledge of the types and amounts of foods specific to their individual dietary needs and activity level. Participants were also required to complete a sports nutrition knowledge survey at the beginning and end of the intervention season.

### 2.2. Subjects

After obtaining approval by the Institutional Review Board for Research with Human Subjects (IRB), 13 participants were recruited, but only 11 completed the study. Two participants were excluded from the study due to separation from the team. The 11 participants were between 19 and 22 years old and were recruited from a NCAA Division I women’s volleyball team (Table 1). The average years of playing competitive volleyball was 6.9 years with a range of 4–11 years. Player positions include outside hitter (*n* = 4), defensive specialist (*n* = 3), middle hitter (*n* = 2) and setter (*n* = 2) with 7 of the 11 being starting players. Each participant was fully informed of the scope and risks of the study prior to signing an IRB approved consent form. 

**Table 1:** Subject characteristics.

	Intervention Season	
	Beginning	End
Age (year)	19.5 ± 1.0	19.8 ± 1.0
Height (cm)	176.3 ± 6.0	176.3 ± 6.0
Weight (kg)	75.4 ± 13.4	76 ± 13.6
Fat Mass (%)	24.5 ± 5.9	22.7 ± 5.6 *
Fat-Free Mass (%)	75.5 ± 13.1	77.3 ± 18.5 *

Values are expressed as mean ± standard deviation. * Significant difference (*p* ≤ 0.05).

### 2.3. Procedures

Participants were instructed to keep a food diary and record all food and beverage ingested for three days in January and April in the 2009 off-season (non-intervention), and each month during the 2010 off-season (intervention). To reflect typical intake and valid results, the three-day food diary consisted of two weekdays and one weekend day. Each participant met with the researcher and was interviewed about food records for accuracy in recording. A computerized nutrient analysis program, Nutrition Data Systems for Research (NDSR, 2009), was used to analyze each of the diet records. This analysis program includes standardized questions to be asked during the interview session to improve accuracy of the food record. Three-day averages for total energy, carbohydrate, protein, and fat were determined for each participant. Body composition was assessed using air displacement plesthysmography using the BOD POD Body Composition System (Concord, CA, USA) following the manufacturer’s recommended procedures. Results from the NDSR were compared to the energy needs estimated using the Nelson Equation: 

RMR (kcal/day) = 25.80 × Fat Free Mass (kg) + 4.04 × Fat Mass (kg) 

which includes lean body mass as a variable and thus may reflect an active individual’s resting energy needs [22].

An individualized nutrition intervention was conducted for each participant based on their NDSR results. The principle investigator, also a Registered Dietitian, met with each participant one week after food diaries were received for a total of four visits during the intervention season. 

At the beginning and end of the intervention season, participants were required to complete the Reilly and Maughan sports nutrition questionnaire to assess each participant’s individual knowledge of sports nutrition [2]. The questionnaire was divided into 10 sections as follows: demographics, eating patterns and dietary behaviors, hydration, weight control, dietary supplements, general nutrition, sports nutrition, protein, strategies for training and food choices, and a swimmers section. Participants were required to complete sections 1 through 9 of the questionnaire with the 10th section being omitted due to its application applying only to swimmers. Question order was changed for the second administration of the questionnaire. The Cronbach α reliability estimate was 0.68 for the population in this investigation.

### 2.4. Statistical Analysis

Paired *t*-tests were performed comparing non-intervention to intervention season as well as pre and post values in the intervention season for energy intake and energy needs; protein intake and protein needs; carbohydrate intake and carbohydrate needs; dietary fat intake and dietary fat needs as well as correct answers on the nutrition knowledge test. All data were analyzed using SPSS version 17.0 (SPSS Inc., Chicago, IL, USA, 2007) with significance set at *p* ≤ 0.05. 

## 3. Results

Dietary collection from the non-intervention season and the intervention season revealed that participants did not meet the recommended energy requirement of 37–41 kcal/kg of body weight. For the intervention season, the average percent of energy intake for the team at the beginning of the season was 56% of estimated needs with a range of 25% to 88%. The average percent of energy intake for the team at the end of the intervention season was 70% of estimated needs with a range of 44% to 95%, representing a significant improvement (*p* = 0.002) (Table 2).

**Table 2:** Female volleyball players (*n*=11) intervention season beginning and end mean intakes of total energy and macronutrients.

		Intervention		Recommendation	*p* value
		Beginning	End		
Energy Expenditure (kcal)		3162 ± 421.3	3162 ± 421.3		
				
Energy Intake (kcal)		1756.0 ± 557.5	2178.4 ± 491.8 *		
	kcal/kg	24.0 ± 8.6	29.4 ± 7.5	(37–41 kcal/kg)	0.002
	% total kcal needs	56.3 ± 18.5	70.0 ± 17.7		
				
Carbohydrate Total (g)		224.3 ± 64.4	304.0 ± 79.9 *		
	g/kg	3.08 ± 1.1	4.15 ± 1.3	(6–10 g/kg)	0.01
	% total kcal	52.3 ± 8.9	56.0 ± 9.2		
	% total kcal needs	48.2 ± 16.2	66.0 ± 21.7	(474.4 ± 63.2 g/day)	
				
Protein Total (g)		69.3 ± 26.8	84.0 ± 20.5 *		
	g/kg	0.9 ± 0.3	1.14 ± 0.3	(1.2–1.7 g/kg)	0.01
	% total kcal	15.5 ± 3.4	15.6 ± 2.9		
	% total kcal needs	59.0 ± 22.2	72 ± 19.8	(118.5 ± 15.8 g/day)	
				
Fat Total (g)		67.4 ± 27.8	69.0 ± 24.8		
	% total kcal	33.7 ± 6.4	27.9 ± 5.2		
	% total kcal needs	77.0 ± 30.5	79.0 ± 26.6	(87.7 ± 11.7 g/day)	0.63

Values are expressed as mean ± standard deviation. * Significant difference (*p* ≤ 0.05).

Based on the dietary collection for the non-intervention and intervention seasons, the team’s average percent of carbohydrate intake did not meet the recommended intake of 6 to 10 g of carbohydrate per kilogram of body weight [14]. It is generally recommended to eat a diet high in carbohydrates at 60% of total energy intake; however, adequate energy intake must be consumed for this recommendation to be appropriate. At the beginning of the intervention season, the team’s average percent of carbohydrate intake was 48% of estimated needs with a range of 29% to 76%. At the end of the intervention season, the team’s average percent of carbohydrate intake was 66% of estimated needs with a range of 33% to 101% representing a statistically significant increase (*p* = 0.01). 

**Figure 1:**
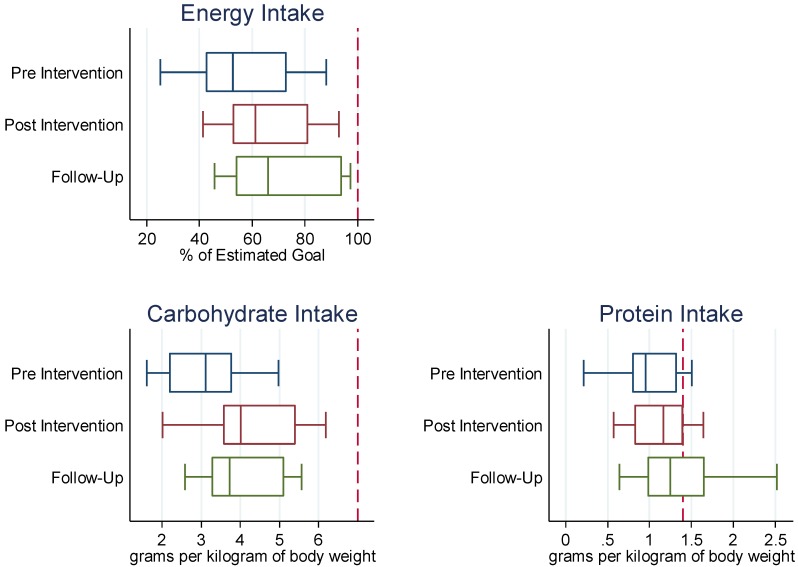
Nutritional intakes at Pre, Post Intervention and at Follow-Up (*N* = 11). Notes: Box plots represent medians, the 25th percentile, and the 75th percentile. Whisker plots represent lower and upper adjacent values. The dashed line represents the (estimated) nutritional goal.

The team’s average percent of protein intake at the beginning of the intervention season was 59% of estimated needs with a range of 16% to 88%. At the end of the intervention season, the average percent of protein intake increased significantly (*p* = 0.01) to 72% of estimated needs with a range of 37% to 102%. At the beginning of the intervention season, the teams’ average percent of fat intake was at 77% of estimated needs with a range of 23% to 124%. At the end of the intervention season, the team’s average percent of fat intake increased slightly to 79% of estimated needs with a range of 52% to 118% (*p* = 0.63) (Figure 1). 

A significant improvement (*p* = 0.001) was seen in the team’s nutrition knowledge after the intervention. Every participant answered more questions accurately after the intervention than prior to the intervention on the sports nutrition knowledge survey. The mean pre-test and post-test scores for all female volleyball players were 24.7 (±5.9) and 31.5 (±6.1), respectively, out of a possible 55 points. Scores ranged from 16 to 37 on the pre-test and 22 to 43 on the post-test. 

## 4. Discussion

The present study revealed that many participants failed to meet current energy, carbohydrate, protein, and fat recommendations for physically active females during two off-seasons. However, from the non-intervention season to the intervention season, there were significant differences seen in energy, carbohydrate, and protein intake (Table 3). The mean energy intake for the team at the beginning of the intervention season was 1756 kcal/day (about 24 kcal/kg of body weight) with 0% of participants in energy balance. At the end of the intervention season, the team’s mean energy intake increased to 2178 kcal/day (about 29 kcal/kg of body weight) with 18% of participants on energy balance (meeting 93%–95% of estimated needs). While this change in mean energy intake is a significant improvement, it is still less than the recommended 37 to 41 kcal/kg of body weight. Similar findings were reported in a study of female volleyball players in Greece, with participants baseline mean energy intake during the off-season was estimated at 1541 kcal/day (about 23.8 kcal/kg of body weight per day) [7]. Moreover, these findings are also supported by other studies showing female athletes with low energy intake compared to energy expenditure [5,7,8,11,12]. 

**Table 3:** Percentage of participants either increasing or decreasing dietary intake according to variable upon completion of the study.

Variable	Intervention End Increase	Intervention End Decrease
Energy	73%	27%
Carbohydrate	82%	18%
Protein	73%	27%
Fat	36%	64%
Total	66%	34%

Values are expressed as mean percentages. Total expressed as mean percentage.

These findings of low energy availability are a major nutritional concern, especially considering the high energy demands of off-season training which was evidenced by participants mean predicted energy expenditure at 3162 kcal/day. According to the Academy of Nutrition and Dietetics, female athletes with persistent low energy intake below 2000 kcal/day can lead to weight loss and disruption of endocrine function [14]. Moreover, it has been reported that female athletes with low energy intake also have poor micronutrient intakes [23] and are at risk for a diet deficient in the micronutrients calcium, iron, magnesium, zinc, and B-complex vitamins [24]. 

In addition to low energy intake, the team’s mean energy intake from carbohydrates was inadequate for sufficient glycogen repletion both prior to dietary intervention and after dietary intervention. The team’s carbohydrate intake at the beginning of the intervention season averaged at 224 g/day (3.08 g/kg) with 0% of participants meeting estimated needs. At the end of the intervention season, mean carbohydrate intake increased to 304 g/day (4.15 g/kg), with 9% (*n* = 1) of participants meeting estimated carbohydrate needs. 

In addition to an increase in the team’s mean energy and carbohydrate intake, fat intake increased as well although not statistically significant. At the beginning of the intervention, the team’s average fat intake was at 77% of estimated needs (67 g/day) while at the end of the intervention, fat intake increased to 79% of estimated needs (69 g/day);27% of the team’s fat intake exceeded estimated needs by greater than 105% with a range of 108% to 181%. Moreover, most of these players failed to meet adequate carbohydrate intake, which indicates the consumption of high fat foods in place of carbohydrate rich foods. This finding is consistent with other female volleyball players [25] and female soccer, track and swimming athletes [6,7,8]. Generally, fat intake should range from 20% to 35% of total energy intake [14]. Although fat is a valuable source of energy, high fat diets have not been shown to be beneficial for athletic performance especially in the presence of inadequate carbohydrate ingestion [26]. Participants need to reduce their fat intake and increase their carbohydrate intake to meet the foregoing recommendation of 6 to 10 g of carbohydrates per kilogram of body weight.

The team’s average protein intake prior to intervention was at 69 g/day (0.9 g/kg of body weight) while protein intake post intervention was at 84 g/day (1.14 g/kg of body weight). Although these values meet the current DRI for all healthy individuals at 0.8 g/kg, some experts advocate that female athletes should consume protein in levels as high as 1.2–1.7 g/kg for optimal performance [6,15]. Furthermore, none of the participants met their estimated protein needs at the beginning of the study. However, 18% (*n* = 2) of participants were within estimated protein needs (meeting 94%–102% of estimated needs) at the end of the study. 

The sports nutrition knowledge survey revealed participants were least knowledgeable in the areas of weight control, dietary supplements, and general nutrition with greater than 50% of participants answering questions incorrectly most often from these sections. Some of the common questions missed by athletes in this study included those on caffeine and sports performance, functions of vitamins, fat burning foods and supplements as well as supplement regulation and safety which are consistent with the findings of Reilly and Maughan [2]. 

The majority of participants (72.7%) had a positive response towards the importance of good nutrition and sports performance. Moreover, 54.5% of participants described their eating habits as fair with nearly 50% reporting a diet based on a wide variety of different foods. The pre-test survey revealed the Media/Internet/Coach/Trainer (83%) as the number one source of nutrition information, whereas the post-test survey revealed a sports dietitian/nutritionist (62.5%) as the number one source of nutrition information.

According to both the pre-test and post-test survey, 50% of participants reported trying to lose weight. In the pre-test survey, a majority of the participants (63.6%) accurately reported that when long term weight loss is desired, athletes should lose at most one to two pounds of body fat per week. However, 50% of participants were unsure of the daily amount of calories that should be reduced to lead to a weekly weight loss of just one pound. In addition, 72.7% of participants report skipping either breakfast (54.5%) or lunch (18.2%) with only 27.3% of participants report not skipping meals or snacks at all. These responses were also evident in the food diaries with the majority of participants not consuming any food or beverage until early afternoon however, reported waking from sleep between 7:00 and 9:00 a.m. 

Generally, large discrepancies of energy intake seen in dietary records of female athletes of a stable weight have mostly been attributed to under-reporting [27]. However, responses generated from the sports nutrition questionnaire might explain some of the inadequacies seen in the food records of this study. For example, the high amount of participants (50%) trying to lose weight, in addition to the high amount (50%) unsure about the appropriate amount of calories to restrict for weight loss, might explain the low calorie intake demonstrated in this study. While this is only an assumption, other reasons female athletes restrict their energy intake have been identified and include disordered eating behaviors, body image issues, and social influences to stay lean [15,20,28]. According to Hinton *et al.* [17], 62% of female collegiate athletes report a desire to lose weight and will resort to decreasing energy and macronutrient intake. Moreover, Rosen, McKeag, Hugh and Curley [29], found that 32% of female collegiate athletes reported using pathogenic weight control behaviors through laxatives, vomiting, and diet pills, and many of these same women felt these risky behaviors were harmless. This study did not assess weight control behaviors; however, it did indicate that no participants reported using “fat burners” such as Trim Spa, Lipodreme, and Ephedrine. 

As previously mentioned, meal skipping evidently played a role in the inadequacies seen in the food records. According to the questionnaire, the most common reason for skipping meals was lack of time (54.5%). Many athletes face barriers that preclude them from maintaining a steady diet schedule. These barriers include class schedules, work, practice, studying, and making time for family and friends. Future studies should address these barriers and propose solutions to accommodate athletes and their busy schedules. 

In conclusion, the three day food records used in this study appeared to accurately represent intake among this population. Moreover, the individualized nutrition counseling used in the study proved to be beneficial in producing positive changes in dietary intake and nutrition knowledge. While these changes did not result in the athletes reaching their individual goals, the results demonstrated a positive trend toward achieving these goals. The small sample size and omission of male athletes limits the findings; however, these results do provide a pilot investigation to utilize on a larger, more diverse population. More studies should explore whether an individualized or a group approach is more beneficial in producing positive dietary results when providing nutrition education among athletic teams. 

## 5. Conclusions

Although the mean dietary intakes reported in the study did not meet current recommendations for total energy and macronutrients, significant changes were seen from the beginning to the end of the intervention season. The effectiveness of the dietary education employed in this study is evidenced by the significant increase in total energy (*p* = 0.002), carbohydrate (0.01), and protein (0.01) intake among participants in addition to the increase in sports nutrition knowledge (*p* = 0.001). Moreover, the changes in dietary intake were maintained by 66% of participants upon completion of the study (Table 3). These changes indicate that dietary education is useful in improving dietary intake and nutrition knowledge among female athletes. Future education needs to focus on counseling female athletes about meeting appropriate energy requirements according to their activity level as well as consuming adequate carbohydrates for sufficient glycogen storage. Strategies for improving dietary intake might include encouraging athletes to select high carbohydrate foods such as whole grain bread, pasta, rice, and cereal and to choose quality protein sources such as lean meats, poultry, eggs, legumes, and low-fat dairy products. An athlete cannot sustain optimal athletic performance with a low energy intake. Thus, athletes should be advised against skipping meals and should be encouraged to keep healthy snacks available. Athletes might also benefit from traveling tips such as packing healthy snacks and selecting appropriate menu items while dining at restaurants. Furthermore, Registered Dietitians with an expertise in sport nutrition are qualified professionals who should be the primary source for athletes and coaches regarding diet information for their respective programs. 
